# Lignin Recovery from Black Liquor Using Integrated UF/NF Processes and Economic Analysis

**DOI:** 10.3390/membranes13020237

**Published:** 2023-02-16

**Authors:** Manorma Sharma, Patrícia Alves, Licínio M. Gando-Ferreira

**Affiliations:** University of Coimbra, CIEPQPF, Department of Chemical Engineering, Rua Sílvio de Lima, 3030-790 Coimbra, Portugal

**Keywords:** crossflow filtration, fouling, integrated UF/NF process, lignin, mixed matrix membranes, economic analysis

## Abstract

Lignin is a polyphenolic biopolymer present in large amounts in black liquor (BL). This work investigated the recovery of lignin from BL (pre-filtered by ultrafiltration (UF)) by nanofiltration (NF). For the NF tests, laboratory-made mixed matrix membrane (MMM) prepared with 0.1% activated carbon (AC) nanoparticles were used in crossflow filtration mode. The effect of pressure (6–15 bar) and volume reduction (VR) (~65%) were analyzed, and the filtration performance was evaluated in terms of permeate flux, lignin rejection rate, and flux reduction. The lignin rejection rate varied in the range of 67–80% with the pressure, however, the highest increases in flux and rejection were observed at 12 bar, which was found to be the optimum pressure. At a VR of ~65%, the permeate flux decreased by ~55% and the lignin rejection rate increased from 78% to 86%. In addition, an economic evaluation was performed for the preparation of UF and NF MMM. The minimum-to-maximum price range was estimated considering the costs of the laboratory and commercial grade regents. It showed a difference of ~10-fold and ~14-fold for UF and NF membranes, respectively. The results of the laboratory-scale study were used to evaluate the economic feasibility of the process for recovering lignin- and hemicellulose-rich retentate streams.

## 1. Introduction

Lignin is considered to be a biopolymer with high potential due to the presence of several active groups [1]. The most widely available type of industrial lignin is kraft lignin, which accounts for nearly 85% of the total amount of industrial lignin [2]. Kraft lignin is present in large quantities in black liquor (BL), which is a by-product of the kraft pulping process. Conventionally, kraft lignin is used as a fuel to meet the energy needs of the pulp and paper industry. However, the separation of this lignin can open up various valorization opportunities and provide enormous amounts of raw material for biorefineries. It can be used for the production of many valuable biomaterials and biochemicals such as polyurethanes, phenolic resins, absorbents, thermoplastic polymer blends, lignin nanoparticles, etc. [3,4,5,6,7,8].

In addition to lignin, BL also contains inorganic pulping chemicals and polysaccharides (mainly hemicelluloses), making BL a complex mixture. For the fractionation of BL, membrane filtration is one of the most studied processes among the different separation technologies such as acid precipitation (e.g., Lignoboost and Lignoforce lignin) and solvent extraction [9,10,11,12,13,14]. It is an efficient and economical process that does not require any change in the physicochemical composition of BL. It can be used to separate the different components of BL based on differences in their molecular weight, charge, and solubility [15,16]. Different types of membranes, namely polymeric and ceramic, ultrafiltration (UF), and nanofiltration (NF) ones, have been studied for BL fractionation. These membranes have shown good separation performances for different components of BL based on their molecular weight cut-off value (MWCO) or pore size [17,18,19,20].

The separation performance of membranes is greatly influenced by their materials. For example, polymeric membranes, which are economic, provide the higher retention and recovery of lignin and hemicelluloses than ceramic membranes do [19]. However, polymeric membranes have some limitations, namely lower chemical and thermal stability and worse performance due to fouling. These factors increase the economic parameters of the separation process because the membranes need to be cleaned or replaced frequently [21]. These limitations can be overcome to some extent by modifying polymeric membranes with an appropriate treatment or nanomaterials. These modified membranes are commonly referred as nanocomposite membranes or mixed matrix membranes (MMM). 

Despite their various advantages, there are few studies on the use of MMM for fractionation of BL [22,23,24,25,26]. In our previous study, MMM for UF and NF were prepared using ZnO nanoparticles and carbon nanotubes (CNT), respectively [24,25,26]. These membranes showed improvements in filtration and separation efficiency compared to those of simple polymeric membranes (prepared without nanoparticles). The UF membranes prepared with a 0.5% ZnO concentration were used for the filtration of BL, as these membranes showed the best results in terms of flow rate and fouling resistance. The UF was used to concentrate slightly degraded hemicelluloses in the retentate, while allowing lignin and inorganic pulping chemicals to permeate through it [25,26].

In addition, the permeate from the UF process was filtered by prepared NF membranes containing CNTs, which resulted in the good retention of lignin, while the inorganic pulping chemicals and highly degraded biopolymers were allowed to pass through the permeate. However, due to the high cost of CNTs, they were replaced with activated carbon nanoparticles (AC), and the prepared MMM were analyzed to concentrate lignin from the UF permeate [27]. The MMM prepared with 0.1% AC showed better performances than CNT-based membranes do. The optimization of the operating variables, namely temperature (40–60 °C), pressure (12–18 bar), and feed pH (9–13), further improved the efficiency of the process. The NF study accessing the applicability and performance of AC-based MMM was performed only at the laboratory scale with a small filtration cell operated in dead-end filtration mode.

This work was carried out as the continuation of a previous study [27] to upscale the separation of lignin using selected AC-based (0.1%) NF membranes. The study was conducted with an equipment operated in crossflow filtration mode (CFF) using UF as a prefiltration step for the efficient recovery of lignin from eucalyptus-based industrial BL. The feed was recirculated until a desirable volume reduction (VR) was achieved. The integrated UF/NF processes were designed to remove extremely high- and low-molecular-weight impurities, such as polysaccharides, inorganic pulping chemicals, and severely degraded organic components of the biomass in the UF retentate and NF permeate, respectively. In addition, a preliminary evaluation of the economics of preparing MMM for UF and NF processes and their application to the filtration of BL was conducted. The economic analysis of the filtration process was performed to recover a certain mass of hemicelluloses and lignin by UF and integrated UF/NF processes, respectively.

## 2. Materials and Methods

### 2.1. Reagents and Materials

**Figure 1:**
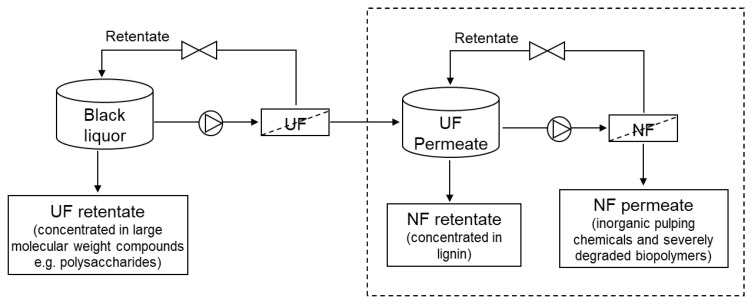
Schematic representation of lignin recovery from black liquor by integrated UF/NF process (only the process represented in dashed box was performed in this study).

Flat sheet AC/PES NF membranes were prepared using 0.1% AC nanoparticles by the nonsolvent (water) induced phase inversion technique as described in our previous work [18]. Industrial kraft BL obtained from eucalyptus wood raw material was prefiltered by using ZnO-based UF membranes. UF was performed in our previous study [25] with the aim of concentrating hemicelluloses in the retentate, and the permeate was used as the feed for the NF (see Figure 1). Therefore, BL in this study refers to the permeate obtained from the UF process. The main characteristics of BL (unfiltered) and BL permeate obtained by UF are reported in Table 1. The methodology adopted for the characterization of BL is described in our previous work [24], except for the hemicellulose analysis. The hemicellulose was analyzed by high-performance liquid chromatography (HPLC), as described in our previous work [25]. The content of hemicellulose reported in the manuscript represents the xylan concentration, since it is the main hemicellulose of eucalyptus species. Its concentration was estimated from the concentration of xylose sugar determined by HPLC.

### 2.2. Nanofiltration Procedure

The Sepa CF II Membrane Cell System and a flat-sheet membrane with a filtration area of 140 cm^2^ were used for NF tests in CFF mode. Based on the optimization results of our previous study [27], 0.1% AC-based PES membranes, filtration temperature, and feed pH were used. A water bath maintained at 60 ± 5 °C was used to maintain the temperature of the feed at 40 °C during BL filtration. The optimized pH in the NF tests was ~11.5, which was achieved by slowly adding concentrated H_2_SO_4_ to the BL while continuously mixing the liquor. Two independent experiments were performed to analyze the effects of transmembrane pressure (TMP) and filtration time (volume reduction). In both of the filtration experiments, permeate samples were collected in predetermined steps, and the retentate was recirculated in the feed tank until a desired concentration was achieved. The schematic representation of the filtration process is shown in Figure 1.

First, a fresh piece of membrane was pressurized in the cell at 16 bar using distilled water as feed. Both the permeate and retentate were recirculated into the feed tank until a stable flux through the membrane was achieved. 

Water filtration tests were then performed to analyze the pure water flux (PWF) and hydraulic permeability of the membranes, as described in our previous work [25]. In brief, NF was carried out along a range of TMPs and at room temperature. The filtration was first performed at 3 bar and continued until 15 bar, and the permeate samples were collected at fixed time intervals. At each TMP, at least 4 permeate samples were collected, and then, the pressure was increased to successively higher values. The PWF (L/m^2^h) was calculated using the permeate volume, effective filtration area of membrane, and the filtration time. Further, plotting the average values of PWF as a function of TMP gave the average permeability of membranes in the tested range of pressure.

After the water filtration tests, BL filtration was performed (in duplicates) to study the effect of the TMP on the permeate flux and retention of lignin and hemicelluloses. After evaluating membrane performance with TMP, BL filtration tests were performed at fixed pressure and temperature values. The effect of volume reduction (VR) or filtration time on permeate flux, flux reduction, and lignin concentration in the retentate and permeate streams was analyzed. The lignin concentration was analyzed by observing the light absorption at 296 nm using a UV/Vis spectrophotometer (T60U, pg instruments, Coventry, UK). For the analysis, a standard calibration curve prepared by plotting lignin concentration as a function of absorption was used. The observed concentrations of lignin in the permeate (C_p_) and feed (C_f_) samples was used for the determination of lignin rejection (1 − (C_p_/C_f_)) [24]. Crossflow velocity (CFV) is an important parameter in CFF and has a positive effect on the membrane performance due to increased turbulence. The influence of CFV on flux and fouling was observed in our previous study conducted with the same equipment and also reported by other researchers in literature [21,25]. However, due to the limitations of the equipment, a CFV of ~3.5 cm/s could only be achieved for the NF membranes.

### 2.3. Economic Analysis of Membranes Preparation and Integrated UF/NF Processes 

**Figure 2:**
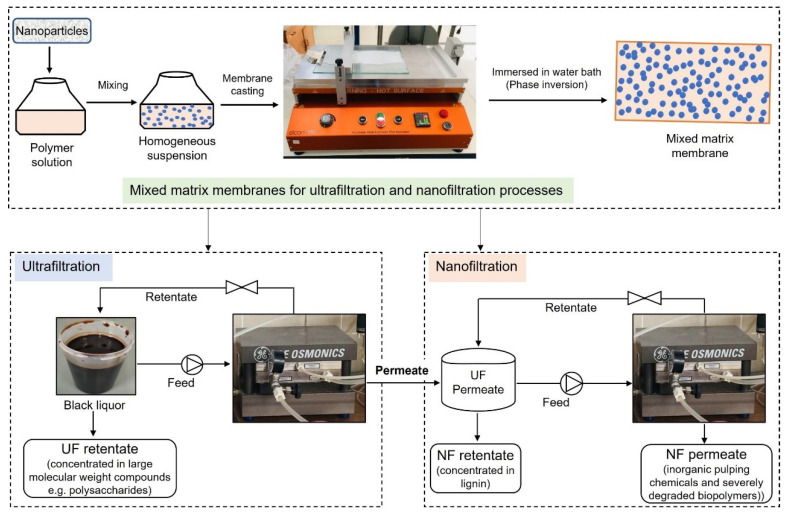
Schematic representation of processes considered for economic analysis.

A preliminary economic analysis was performed for the preparation of the MMM for the UF and NF processes and the filtration of BL with these membranes, which is shown in Figure 2. For this study, the membranes that were used for actual fractionation of BL were considered. The composition of the casting solution used to prepare these membranes is shown in Table 2. These membranes were selected based on various properties such as membrane permeability, hydrophilicity (water contact angle—WCA), BL permeate flux, fouling resistance, and the rejection of lignin and hemicelluloses. 

For calculating the costs, mainly the prices of the consumption of reagents, water, and treatment of the generated effluent were considered. For the reagent costs, two costs were estimated to determine the highest and lowest ranges of the membrane manufacturing costs. For the highest cost estimate, the prices of laboratory grade reagents purchased to perform the work were considered, while the lowest cost estimate was made considering the cost of commercial grade reagents obtained from the internet [28]. For a comparative analysis, the calculated costs were also compared with the purchase costs of commercial UF and NF membranes. Polyethersulfone-based commercial UF (Microdyn Nadir^TM^ UF010, MWCO = 10 kDa) and NF membranes (Microdyn Nadir^TM^ NP010, MWCO = 1–1.2 kDa) were purchased from SterliTech Corporation, Auburn, US and used in our previous work [24].

**Figure 3:**
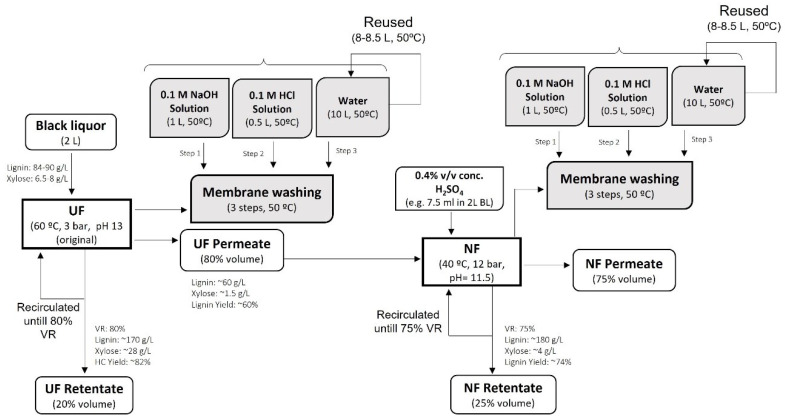
Simplified process flow diagram for BL filtration by integrated UF/NF process with laboratory scale mass balance.

In addition, the economic analysis of the filtration process was performed by considering the cost of the membranes needed for the filtration of BL, the reagents, and the water needed to clean the used membranes. The simplified process diagram with the mass balance at laboratory scale is shown in Figure 3.

Afterwards, the economic analysis for the filtration process was performed by scaling up the process using laboratory data. The required area for UF and NF membranes for the scaled-up process was calculated using Equation (1).

(1) $$\mathrm{Membrane}\;\mathrm{area}\;\left(A_{m}\right)=\frac{\mathrm{Feed}\;\mathrm{flow}\;\mathrm{rate}\;\times \;\mathrm{Volume}\;\mathrm{reduction}}{\mathrm{Average}\;\mathrm{flux}}$$

Due to the limitation of the equipment used in the filtration tests performed in the laboratory, a volume reduction of about 60–65% could be achieved. However, for BL fractionation, reductions of the feed volume to 80% by the UF and 75% by the NF process were targeted (as shown in Figure 3).

**Figure 4:**
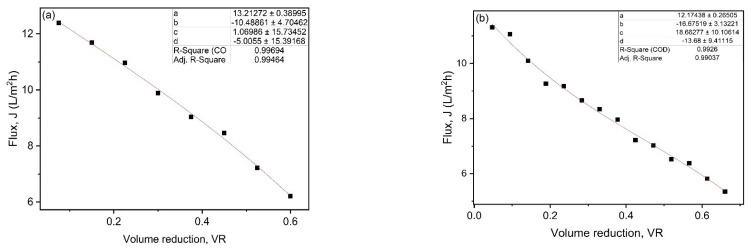
Fittings of permeate flux against VR data for (**a**) UF and (**b**) NF.

The average flux (*J_avg_*) in batch filtration was calculated by integrating the polynomial Equation (2), which represents flux as a function of VR during filtration, leading to Equation (3).

(2) $$J=a+\left(b\times \mathrm{VR}\right)+\left(c\times {\mathrm{VR}}^{2}\right)+\left(d\times {\mathrm{VR}}^{3}\right)$$

(3) $$J_{avg}=a+\left(\frac{b}{2}\times \mathrm{VR}\right)+\left(\frac{c}{3}\times {\mathrm{VR}}^{2}\right)+\left(\frac{d}{4}\times {\mathrm{VR}}^{3}\right)$$

where, a, b, c and d are polynomial coefficients and can be obtained by fitting Equation (2) to the experimental data of flux as a function of VR, as shown in Figure 4a,b for the UF and NF membranes flux data.

To calculate the area of membranes required (using Equation (1)), the feed flow rate was calculated using Equation (4).

(4) $$\mathrm{Feed}\;\mathrm{flow}\;\mathrm{rate}\;=\frac{\mathrm{Lignin}\;\mathrm{or}\;\mathrm{hemicellulose}\;\mathrm{extraction}\;\mathrm{rate}\;\left(\mathrm{kg}/h\right)}{\mathrm{Yield}\;\times \mathrm{initial}\;\mathrm{Concentration}\;\left(\mathrm{kg}/m^{3}\right)}$$

where the extraction rates were assumed to be as 100 kg/h for hemicelluloses by ultrafiltration and 1000 kg/h for lignin by nanofiltration process. The initial concentration and yield were determined using laboratory data of BL characterization, lignin retention, and VR. 

## 3. Results and Discussion

### 3.1. Determination of Membrane Permeability (L_p_)

A preliminary characterization of the membranes based on water filtration tests facilitated the determination of hydraulic permeability. The selected NF membranes (0.1% AC-based membrane) had an average permeability of 3.19 ± 0.54 L/(m^2^ h bar). The membranes were compressed at 16 bar for 1 h to obtain a uniform flux before the permeability evaluation was performed. These membranes showed substantially lower permeability compared to the permeability measured in the dead-end filtration mode [27]. This can be attributed to the low CFV value due to limitation of the used equipment. The same membranes were used for the BL filtration tests after assessing the filterability of the water.

### 3.2. Effect of Pressure on Nanofiltration of BL

**Figure 5:**
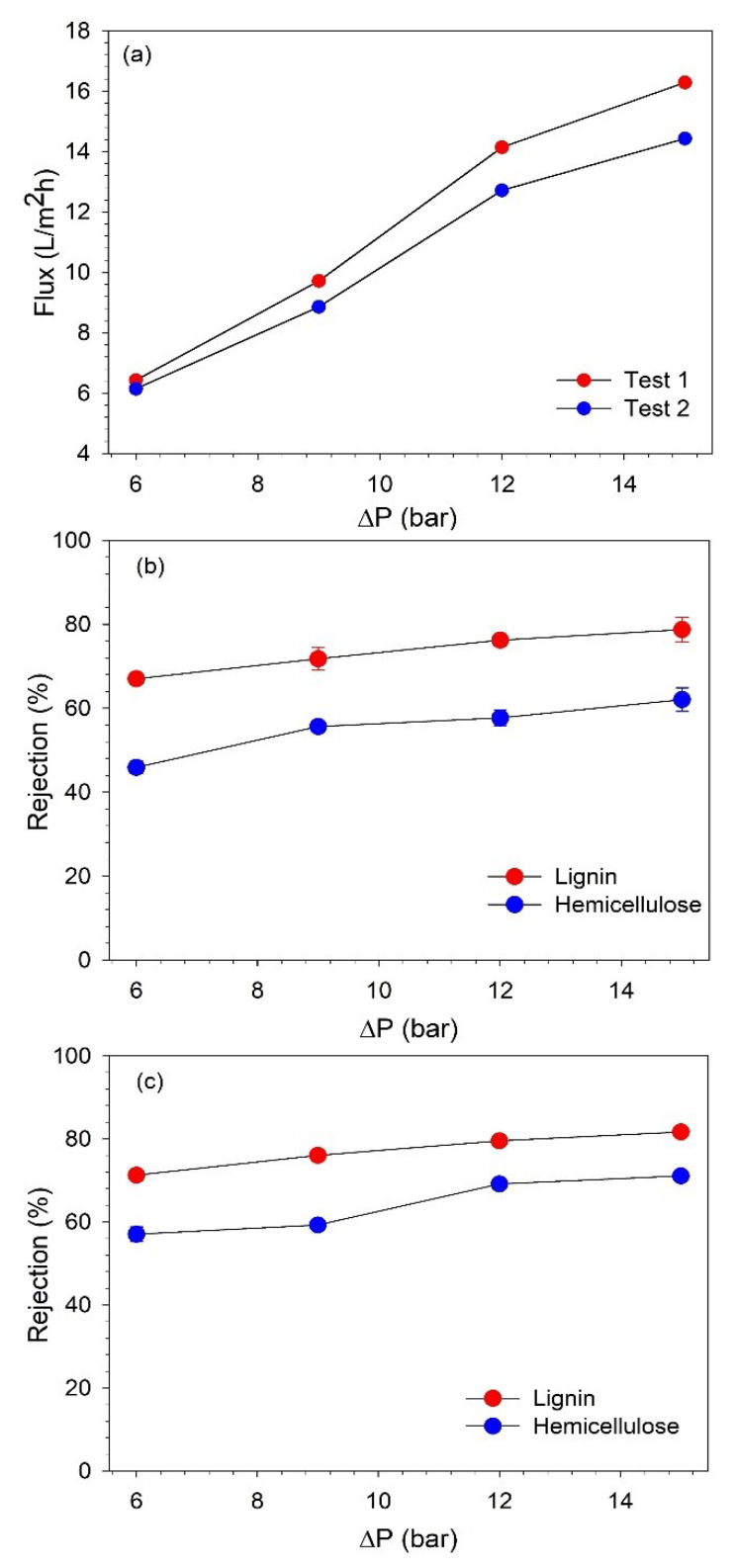
(**a**) Variation of BL permeate flux with TMP and rejection of hemicelluloses and lignin along TMP in filtration (**b**) test 1 and (**c**) test 2.

After the initial characterization, the effect of TMP on the BL permeate flux and rejection of lignin and hemicelluloses was investigated. The pressure was varied from 6 to 15 bar. The pressure range studied in this work was lower than that which was studied for filtration in the dead-end mode. This can be justified since the equipment could not operate at a higher TMP, as increasing the pressure beyond 15 bar resulted in very strong turbulence in the system. The filtration was started at 6 bar, and after the sample collection, the pressure was increased to subsequently higher values. At each pressure, the flow was first stabilized for 10 min, and then two permeate and one retentate samples (before changing to the next pressure) were collected. The samples were analyzed for the concentrations of lignin and hemicellulose to evaluate the rejection of these components. The filtration test was performed in duplicates, and they are presented as test 1 and test 2. The effect of TMP on BL permeate flux and the rejection of lignin and hemicellulose during both of the filtration tests is shown in Figure 5.

It can be observed that the permeate flux increased with an increasing TMP, and the highest flux was obtained at 15 bar. However, after 12 bar, the flux started to stabilize as the flux increased by 45.59% and 43.55% in tests 1 and 2, respectively, when the pressure was increased from 9 to 12 bar. While when the pressure was further increased to 15 bar, the flux improved only by 15.15% and 13.48% in tests 1 and 2, respectively. This indicates that with the used filtration equipment and at the studied operating conditions, 12 bar is the optimum pressure to obtain high permeate flux. As it was observed, further increasing the pressure beyond 12 bar does not lead to a proportional improvement in flux [29].

The concentrations of lignin and hemicelluloses were analyzed to determine the rejection of these components. The retention of lignin and hemicelluloses also increased with an increasing TMP, as shown in Figure 5b,c. The increase in the retention of lignin and hemicelluloses was more pronounced up to 12 bar, and then, it started to stabilize. This confirms that optimum performance can be achieved at 12 bar. The lignin retention varied at 67–82%, while hemicellulose retention changed in the range of 45–71% when the pressure was changed from 6 to 15 bar. Therefore, additional NF tests were performed at 12 bar to concentrate the lignin to a desired VR.

### 3.3. Effect of Volume Reduction on Membrane Fouling and BL Concentration

**Figure 6:**
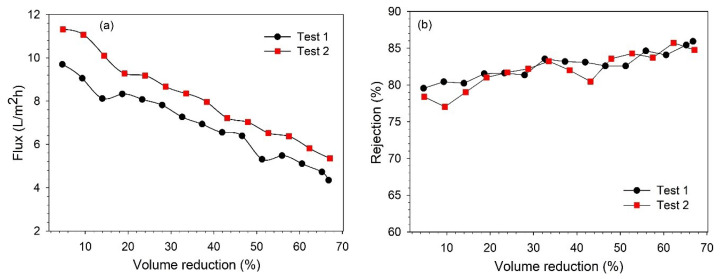
Influence of VR over (**a**) permeate flux; (**b**) lignin rejection rate.

The BL concentration studies were performed at 12 bar to concentrate the lignin in the NF retentate. Filtration was performed by recirculating the retentate in the feed tank. The permeate and retentate samples were collected to analyze the changes in the lignin concentration. The feed was concentrated to ~65% VR, and the filtration took about 9.5–11 h to complete. The higher VR could not be achieved due to the limitation of the equipment. These NF tests were performed without any membrane cleaning until the desired VR (~65%) was achieved. The variations in the permeate flux and lignin retention along the VR are shown in Figure 6.

**Figure 7:**
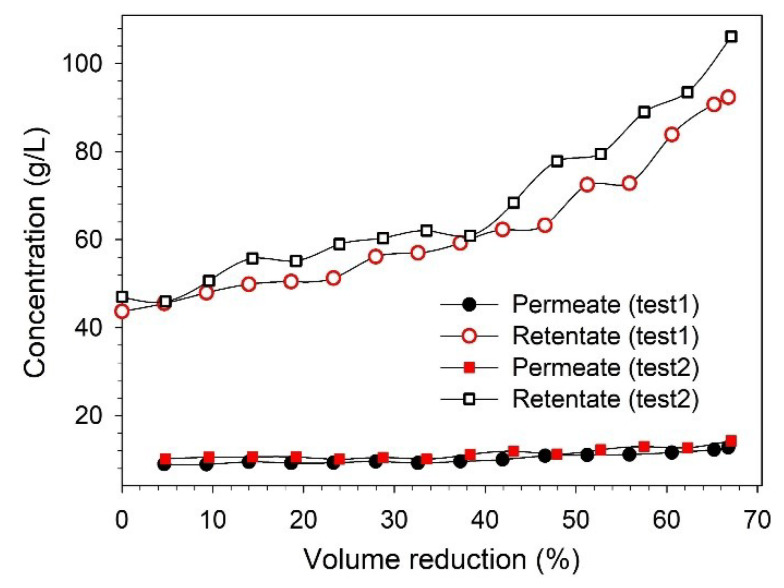
Influence of VR over lignin concentration in permeate and retentate streams.

It was observed that the permeate flux decreased continuously during the filtration time, as a mutual effect of changes in the feed concentration and fouling of the membrane [26,30]. At the end of filtration (66–67% VR), the permeate flux decreased by 53–55%. From Figure 6b, it can be observed that the lignin retention also increased with filtration time, and the retention varied in the range of 78–86%. The increase in retention over the filtration time can be explained as an effect of membrane fouling and increasing feed concentration, which was also observed in our previous study [25,27]. As mentioned earlier, both the permeate and retentate samples were collected during filtration to analyze the variation in the concentration along the VR. The variations in the lignin concentration in the permeate and retentate along the VR are shown in Figure 7. It was observed that the lignin concentration in the final retentate at ~65% VR increased more than two-fold compared to the concentration in the feed. Additionally, the separation of highly degraded biopolymers and inorganic pulping chemicals was noted in the permeate stream by analyzing the composition of BL samples in terms of total dissolved solids, organic, and inorganic contents (see Figure S1).

The xylose concentration in the feed (~1 g/L) was lower when it was compared with the lignin concentration (~45 g/L), along with excretion, which was also low. Therefore, the xylan concentration was analyzed only in the feed and in the initial and final samples of permeate and retentate. In the final retentate, the lignin and xylose concentrations were approximately 106 g/L and 2 g/L, respectively. However, due to various limitations imposed by the equipment, it was not possible to achieve a higher VR than ~65% during the lignin concentration study. This could be improved in the future to obtain a more lignin-concentrated retentate.

### 3.4. Economic Analysis for Preparation and Application of MMM for BL Fractionation

As mentioned in the previous section, the economic analysis of the preparation of MMM considers only the costs of reagents, utilities such as water, and treatment of generated effluent. The costs of the equipment and power for the stirring and mixing of solutions needed at the target scale were not considered. Two different types of reagent costs were considered for the cost evaluation: (a) the laboratory-grade reagents purchased at the beginning of the membrane preparation work and (b) the cost of the industrial-grade (commercial) reagents, which were obtained from specialized websites [28]. Based on these two types of costs, the minimum and maximum price ranges for the preparation of UF and NF mixed matrix membranes were estimated. The costs for both types of reagents, which were considered for the economic analysis, are shown in Table 3. In the laboratory, a membrane with an area of 15 cm × 30 cm (450 cm^2^) could be prepared and considering these dimensions, the cost of membrane preparation was determined. This cost was used to estimate the cost per m^2^ area.

For the casting of laboratory-scale membranes, 2.5 L of distilled water was used as a non-solvent and 9 L of water was used for washing (for three samples of membranes). This resulted into the generation of a total of ~5.5 L of effluent in the production of a membrane with an area of 450 cm^2^. 

**Figure 8:**
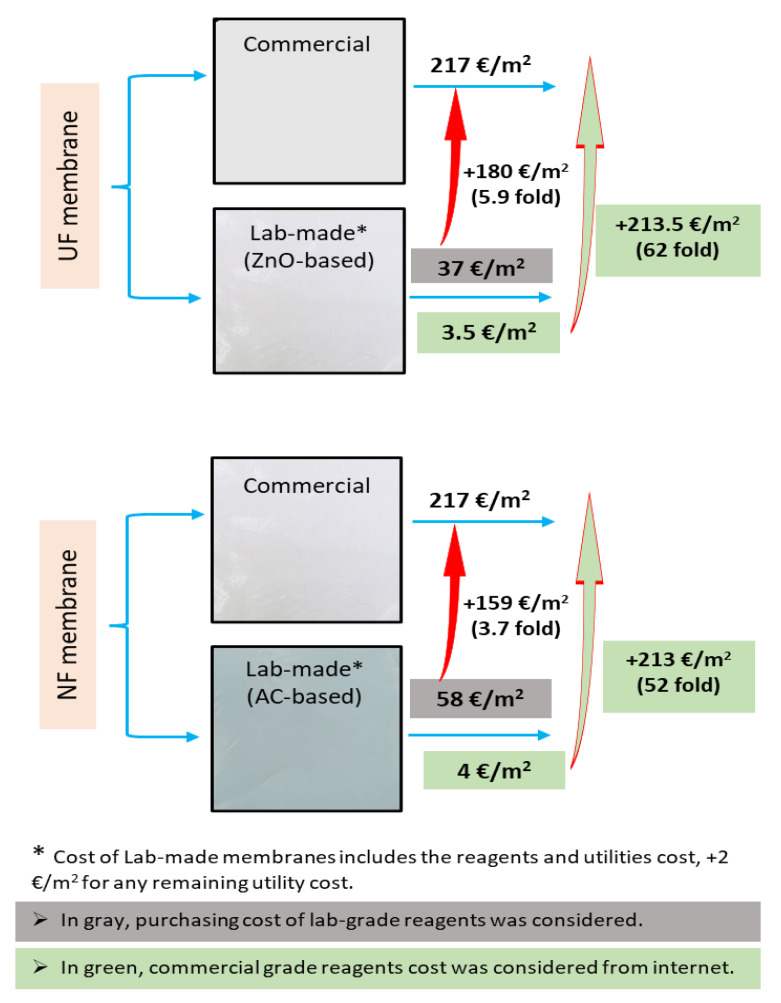
Comparative analysis of membrane preparation cost with commercial membranes prices.

Taking into account the commercial grade prices of all of the components of the membranes, the costs of producing 0.5% ZnO-based UF and 0.1% AC-based NF membranes, are EUR 1.5/m^2^ and EUR 2.2/m^2^, respectively. In contrast, when laboratory-grade reagent prices were considered, the membrane costs increased ~23-fold and ~25-fold per m^2^ area, respectively. Since the cost of electricity and any other ancillary costs were not included in these prices, EUR 2/m^2^ was added to the calculated cost of membrane production to compensate for the remaining ancillary costs. As mentioned above, a comparative cost analysis was also performed for the laboratory-scale production of these membranes with the purchase costs for commercial polymeric membranes, which is shown in Figure 8. 

Additionally, the prepared MMM showed good resistance to fouling, and most of the permeate flux was recovered during the chemical cleaning process. Approximately 96% and 98% of the original hydraulic permeability of 0.5% ZnO-based UF and 0.1% AC-based NF membranes were recovered by cleaning [25,26,27]. Therefore, these membranes represent a good opportunity for their reuse. Further, in addition to the cost estimation for the fabrication of new membranes, an economic evaluation of the chemical cleaning process was also performed. In the chemical cleaning process, the membranes used for BL filtration were successively cleaned in three steps with 0.1 M NaOH, 0.1 M HCl solutions and distilled water (see Figure 3). This chemical cleaning process incurred costs of ~ EUR 1/m^2^ when we were using commercial grade reagents and ~EUR 8/m^2^ using laboratory grade reagents.

The prepared membranes were used for the fractionation of BL to separate hemicellulose- and lignin-rich retentate streams from the UF and NF processes. A proposed flow diagram of the integrated UF/NF system for BL filtration is shown in Figure S2. However, for the economic analysis of the overall process, it was assumed that all of the equipment and configurations are already in place. The membrane area required was calculated by assuming extraction rates of 100 kg/h hemicellulose in the UF process and 1000 kg/h lignin in the NF process. The total areas for UF and NF membranes to meet the assumed extraction rates were 1410 m^2^ and 2110 m^2^, respectively. 

The total cost of the required membranes was calculated based on the cost of membrane preparation calculated by considering the cost of commercial grade reagents (see Figure 8). The cost of UF membranes for BL filtration in a batch process was estimated to be about EUR 250/m^3^. Moreover, this process, together with the targeted hemicellulose extraction, will lead to the extraction of 700 kg/h of high-molecular-weight lignin. On the other hand, the NF cost about EUR 316/m^3^ for the filtration of 27 m^3^ of permeate from the UF process to achieve an extraction of about 1000 kg/h of lignin. 

However, only a preliminary economic analysis was performed in this study. A more comprehensive economic analysis will be an interesting topic of work for the future. A complete economic analysis will involve the estimation of both the operating costs and capital investment to separate a desired amount of lignin and hemicelluloses from BL. Additionally, it will be interesting to study BL fractionation by considering the design of different modules suitable for industrial scale based on the formulation used in the preparation of flat sheet membranes. These aspects would be helpful to explore the feasibility of using these MMM for the fractionation of BL at an industrial scale. 

## 4. Conclusions

In this work, the study of lignin recovery from kraft BL was performed by employing an NF process using an AC-based PES membrane. UF with ZnO-based MMM was used in a prefiltration step. The filtration was performed in a batch process to achieve a desirable volume reduction (VR) in the crossflow mode. First, the effect of the transmembrane pressure on permeate flux and the rejection of hemicellulose and lignin was analyzed. During the filtration process, the highest improvements in flux and rejection were observed at 12 bar, which was found to be the threshold pressure. Moreover, filtration was performed at 12 bar for a VR of ~65%, which improved the lignin concentration in the final retentate by more than two-fold. 

The analysis of the economic aspects of the cost of membrane preparation for UF and NF processes was performed considering the cost of the commercial and laboratory grade reagents. It was found that the cost of the preparation of UF and NF membranes will be in the range of between EUR 3.5–37/m^2^ and EUR 4–58/m^2^, respectively. The economic study indicated also that the preparation of these mixed matrix membranes will result in a cost savings compared to the cost of purchasing commercial UF and NF membranes. Since the MMM produced in the laboratory allowed us to achieve the complete recovery of the flux after cleaning, there was also the possibility of a cost saving by reusing the prepared membranes.

## Figures and Tables

**Table 1:** Physicochemical characteristics of industrial BL (unfiltered) and permeate obtained from the UF process.

Parameter	BL (Unfiltered)	BL (UF Permeate)
pH	12.92 ± 0.41	13.03 ± 0.26
Density (kg/m^3^)	1092.64 ± 0.40	1090.49 ± 3.10
Total Solids (% *w*/*w*)	16.99 ± 1.04	14.57 ± 0.34
Lignin (g/L)	84–92	36–40
Hemicelluloses (g/L)	6.5–8	0.7–1

**Table 2:** Composition of casting solutions used for mixed matrix UF and NF membranes.

Components	UF Membranes	Amount (%*w*/*w*)	NF Membranes	Amount (%*w*/*w*)
Base polymer (polymer)	Polyether sulfone (PES)	12	PES	18
Pore forming agent (PFA)	Polyethylene glycol-1500 (PEG)	3	Polyvinyl pyrrolidone (PVP)	1
Nanoparticles (NPs)	Zinc oxide (ZnO)	0.5	Activated carbon (AC)	0.1
Solvent	Dimethylacetamide (DMAc)	84.5	DMAc	80.9

**Table 3:** Cost of reagents and utilities used for preparation of UF and NF membranes.

Components	Units	Commercial Grade	Laboratory Grade
DMAc	EUR/kg	2.64	42.13
PES	(EUR/kg)	16.11	556.00
PEG	(EUR/kg)	3.24	38.40
ZnO	(EUR/kg)	2.63	35.91
PVP	(EUR/kg)	18.80	591.00
AC	(EUR/kg)	244.40	6500.00
NaOH	EUR/kg	0.72	8
HCl	EUR/L	0.5	4.6
Water	EUR/m^3^	0.22	
Effluent discharge	EUR/m^3^	0.35	

## Data Availability

The data presented in this study are available upon reasonable request from the corresponding author.

## Supplementary Materials

The following are available online at https://www.mdpi.com/article/10.3390/membranes13020237/s1, Figure S1: Composition of kraft black liquor samples observed before filtration and after UF and NF processes. Figure S2: Process flow diagram for integrated UF/NF process implemented for separation of hemicelluloses and lignin from black liquor.
