# Supplementary material for: Lignin Recovery from Black Liquor Using Integrated UF/NF Processes and Economic Analysis
Source: Membranes (Basel). 2023 Feb 16;13(2):237. doi: 10.3390/membranes13020237 (PMC9961576; doi:10.3390/membranes13020237)
Supplement: Supplementary file 1 [file membranes-13-00237-s001.zip › membranes-2209222-supplementary.pdf]

*membranes*

*Supplementary material*

# Lignin Recovery from Black Liquor Using Integrated UF/NF Processes and Economic Analysis

**Manorma Sharma *, Patrícia Alves and Licínio M. Gando-Ferreira ***

University of Coimbra, CIEPQPF, Department of Chemical Engineering, Faculty of Sciences and Technology, Pólo II, Rua Sílvio Lima, 3030-790 Coimbra, Portugal
* Correspondence: manorma@eq.uc.pt (M.S.); lferreira@eq.uc.pt (L.M.G.-F.)

## 1. Composition of black liquor samples

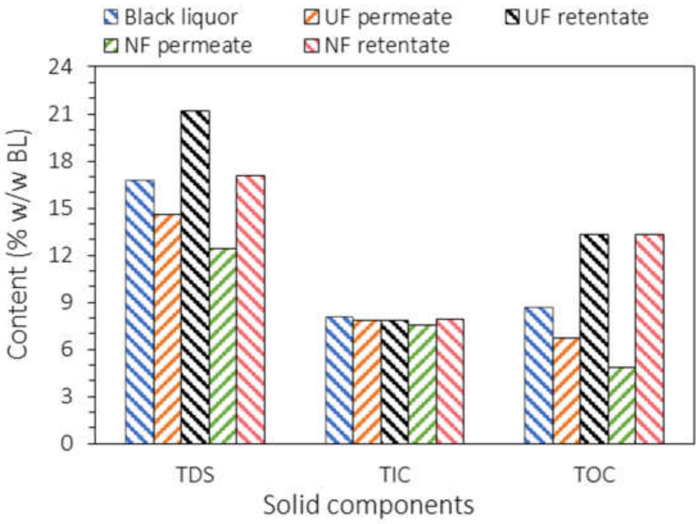


**Figure S1.** Composition of kraft black liquor samples observed before filtration and after UF and NF processes.

## 2. Proposed process flow diagram for integrated UF/NF process

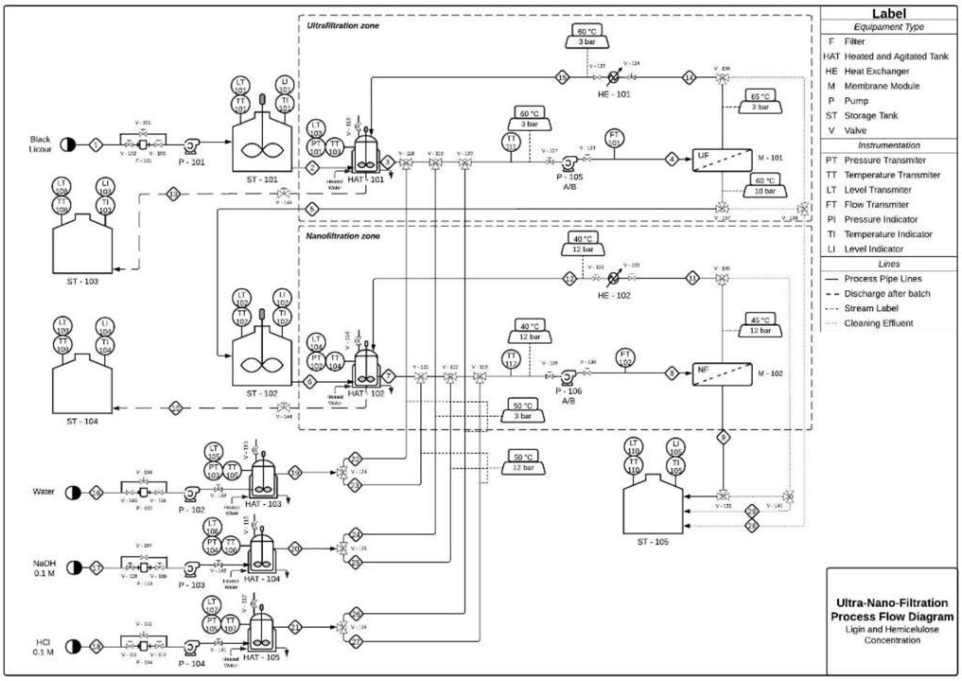


**Figure S2.** Process flow diagram for integrated UF/NF process implemented for separation of hemicelluloses and lignin from black liquor.
